# Complete Genome Sequences of Mycobacteriophages Balomoji, Crumble, Frederick, ShowerHandel, TinyTimmy and Zabiza

**DOI:** 10.17912/micropub.biology.001478

**Published:** 2025-06-23

**Authors:** Zachery Middleton, Giovanna Alves, Em Bavuso, Phuong-Anh Bui, Kai Jandial, Drew Kirchner, Taylor Kiskamp, Blair Morgan, Kezie Osei, Rohit Paradkar, Sofia Taylor, Allison A. Johnson

**Affiliations:** 1 Virginia Commonwealth University, Richmond, Virginia, United States; 2 Center for Biological Data Science, Virginia Commonwealth University, Richmond, Virginia, United States

## Abstract

We report the genome sequences of six novel phages infecting *Mycobacterium smegmatis* mc ^2^ 155. Each phage has siphoviral morphology and a double-stranded DNA genome. These genomes represent five different clusters of Mycobacterium phages, including a cluster K1 phage with a genome deletion that impacts the genes involved in lysogeny.

**Figure 1. Plaque morphology and genome context of mycobacteriophage Zabiza:**
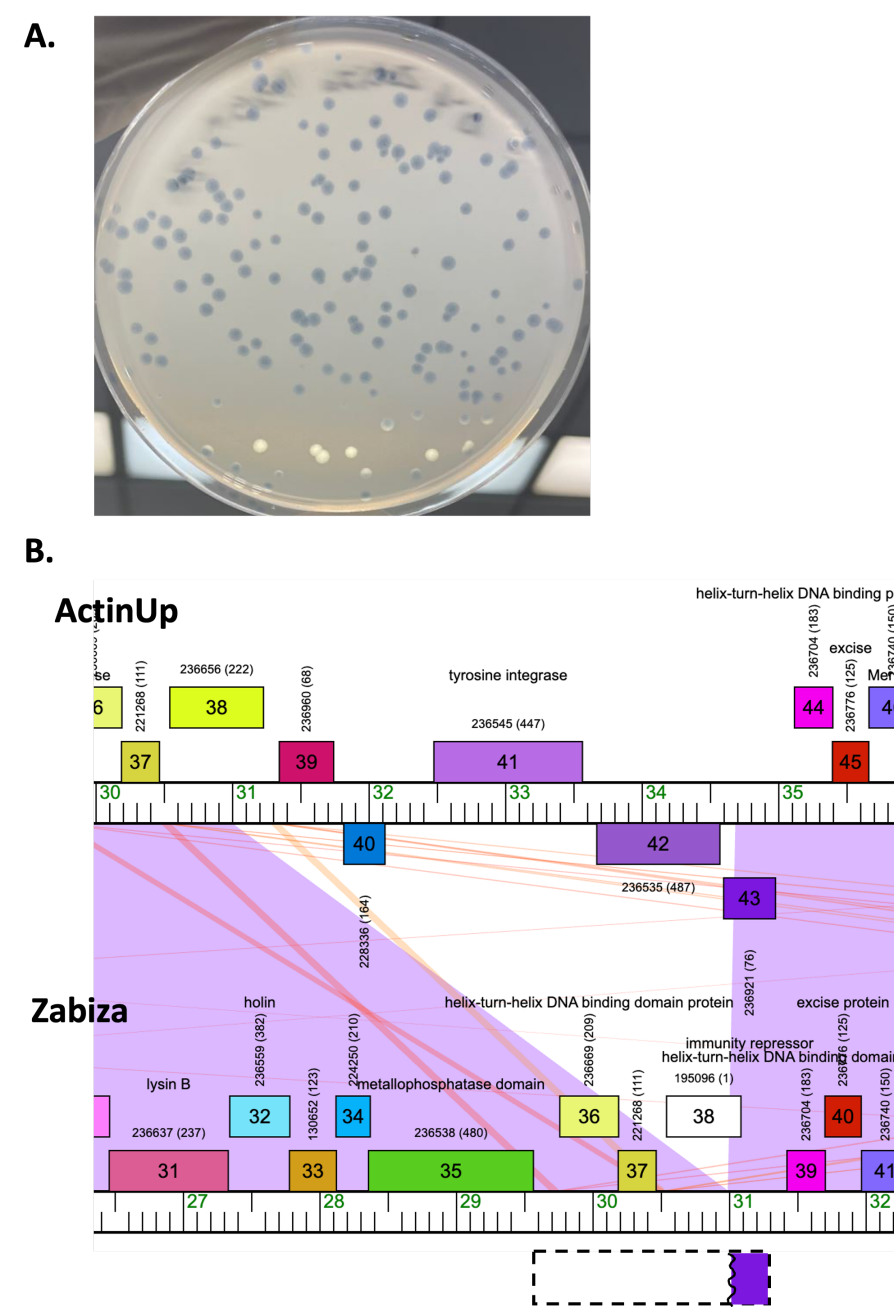
A) Plaque morphology of mycobacteriophage Zabiza shows clear plaques with no halo. B) Genome map region of cluster K1 mycobacteriophages ActinUp and Zabiza. The genome regions encoding the integrase and immunity repressor in ActinUp and the corresponding region in Zabiza are shown, with alignment of the genomes by cro and excise proteins. Protein coding genes are indicated by colored rectangles. Proteins with predicted function are labeled. The dotted line rectangle represents a large open reading frame in Zabiza, with a purple region that matches the first 97 amino acids of the 126 amino acid repressor protein from ActinUp (ActinUp gp43). The Zabiza ORF lacks a corresponding stop codon and continues until 29523. Purple and pink shading between the genomes indicates homologous regions with high percent identity and low e-values in blastn.

## Description

Discovery of mycobacteriophages advances our understanding of phage biology. Mycobacteriophage infect and lyse *Mycobacterium* hosts. With the increasing prominence of phage therapy as treatment for antibiotic-resistant nontuberculous mycobacterium infections (Dedrick et al., 2023) understanding of phage genome diversity can provide invaluable insights. This article describes the discovery and genome characteristics of six *Mycobacterium* phages.

Phage were isolated from soil samples (Table 1) and characterized through established methodologies (Zorawik et al., 2024). Samples were extracted in liquid medium (Middlebrook 7H9 supplemented with 145 mM NaCl, 5% Albumin fraction V, 2% dextrose and 1 mM CaCl _2_ ), the wash filtered (0.22 um pore), and the filtrate either directly plated in top agar containing *M. smegmatis* mc ^2^ 155 (Frederick) or first inoculated with *M. smegmatis* and incubated at 37˚C with shaking for 24-48 hr to enrich for mycobacteriophages before being re-filtered and plated in top agar. Phage were purified by several rounds of plaque assays. Then, a high titer lysate was prepared by flooding a plate with a high density of plaques with phage buffer (10 mM Tris pH 7.5, 10 mM MgSO _4_ , 68 mM NaCl, 1 mM CaCl _2_ ). Negative staining (1% uranyl acetate) and transmission electron microscopy revealed siphoviral morphologies for all the phages. (Table 1).

DNA was isolated from phage lysates using the Promega Wizard DNA cleanup kit. Genomic DNA for phage Frederick was sequenced by 454 FLX sequencer following library preparation using GS FLX Titanium series reagents whereas genomic DNA for all other phages were sequenced by Illumina MiSeq sequencer (v3 reagents) following library preparation using NEBNext Ultra II FS kit. Reads were assembled into a single contig for each genome using Newbler (v1.1 for phage Frederick, v2.9 for all other phages). Genomes were visually checked for end type and completeness using Consed (v20 for phage Frederick or v29 for all other phages) ^3^ . Sequencing and genome details are summarized in Table 1.

To annotate the genes in each genome, DNA Master ( http://cobamide2.bio.pitt.edu ) or PECAAN (Reinehart et al., 2016), annotation platforms that integrate data from genomic databases and bioinformatics tools, were used. Open reading frames (ORFs) were predicted by Glimmer v3.02 (Salzberg et al., 1998) and Genemark v2.5 (Besemer & Borodovsky, 2005), and tRNA genes were predicted by Aragorn v1.2.38 (Laslett & Canback, 2004) and tRNAScan v2.0 (Lowe & Chan, 2016). Predicted protein functions were determined using HHPred (PDB_mmCIF70, Pfam-A, NCBI_Conserved_Domains databases (Söding et al., 2005)), Blastp (NCBI non-redundant database (Altschul et al., 1997)), and DeepTMHMM (Hallgren et al., 2022). Default settings were used for all software. Positional and functional annotations for each gene were manually curated (Table 1). Phages were assigned to clusters based on gene content similarity of at least 35% to phages in the Actinobacteriophage database, https://phagesdb.org/ (Table 1) (Hatfull et al., 2010; Russell & Hatfull, 2017; Gauthier & Hatfull, 2023).

Based on identifiable integrase and immunity repressor gene functions, phages Balamoji, Crumble, and ShowerHandel are predicted to be temperate. Experimental studies will be necessary to demonstrate lysogeny. Genes typically involved in lysogen formation were not identified in Frederick, and TinyTimmy, which are thus unlikely to establish lysogeny. Cluster K1 phage Zabiza genome contains a deletion that impacts integrase and repressor, while retaining excise and cro. The deletion results in the absence of the tyrosine integrase (Figure 1). The Zabiza genome has also retained a region with coding potential and 100% homology to 78% query coverage of ActinUp repressor protein, but lacks a corresponding stop codon. Wet lab experiments would be needed to confirm whether this repressor is functional. This is an important observation since phages in this predominantly temperate cluster have been shown to infect both fast- and slow-growing mycobacterial hosts (Pope et al., 2011). Examination of 115 cluster K1 mycobacteriophages (Russell and Hatfull, 2017) revealed three other cluster K phages that appear to lack an identifiable integrase, and would be predicted to not form lysogens. Among the 641 total putative ORFs identified across all 6 phage genomes, two are novel genes not found in the Actinobacteriophage database. These novel genes lack predicted function. Finally, Crumble and ShowerHandel encode mycobacteriophage mobile elements (MMPE1 and MMPE2, respectively), which are recalcitrant and found in distinct regions across related phage genomes (Sampson et al., 2009).

Data availability

GenBank and Sequence Read Archive (SRA) accession numbers are provided in Table 1.

**Table:** 

**Table 1. Phage and genome sequence characteristics**						
Phage	**Balomoji**	**Crumble**	**Frederick**	**ShowerHandel**	**TinyTimmy**	**Zabiza**
Sample Location (City, State)	Richmond, VA	Richmond, VA	Richmond, VA	Richmond, VA	Richmond, VA	Richmond, VA
Sample location GPS coordinates	37.54572 N, 77.44931 W	37.544917 N, 77.455 W	37.545955 N, 77.453922 W	37.544897 N, 77.454942 W	37.5435 N, 77.4478 W	37.54574 N, 77.45141 W
Predicted lifestyle	Temperate	Temperate	Lytic	Temperate	Lytic	May be lytic
Plaque Morphology	clear with halo	clear	clear	clear	clear	clear with halo
Plaque Size	2.5 ± 0.8 mm (n = 10)	1.7 ± 0.7 mm (n = 10)	1 mm (n=1)	1.2 ± 0.2 mm (n = 10)	0.9 ± 0.2 mm (n = 10)	2.9 ± 0.6 mm (n= 10)
Capsid Size (nm)	61.8 ± 1.2 nm (n = 3)	49.3 ± 2.4 nm (n = 3)	54 nm (n=1)	61.8 ± 1.2 nm (n = 3)	63.2 ± 1.2 nm (n = 3)	52.8 ± 2.4 nm (n = 3)
Tail Length (nm)	254.9 ± 7.9 nm (n = 3)	144.4 ± 2.4 nm (n = 3)	175 nm (n=1)	198.6 ± 3.2 nm (n = 3)	138.9 ± 6.0 nm (n = 3)	179.2 ± 2.1 nm (n = 3)
SRA accession number	SRX25734218	SRX25734219	SRX25734228	SRX25734221	SRX25734223	SRX25734225
# of reads	742,315	291,815	6,185	163,063	3,790,164	2,692,655
Approximate read coverage	1420	717	34	400	15804	11874
Genbank accession number	PP978826	PP978871	PQ244021	PP978878	PQ362662	PQ362688
Cluster	E	F1	B4	F1	A11	K1
Genome length (bp)	76419	53424	58937	57562	51840	56117
# ORFs (# with predicted function)	144 (50)	99 (40)	98 (40)	107 (42)	97 (42)	91 (51)
# Orphams	1	0	0	0	0	1
# tRNAs	2	2	0	0	1	1
GC content (%)	62.8	61.4	61.4	61.4	63.7	66.7
End type	Defined end	Defined end	Circularly permuted	Defined end	Defined end	Defined end
Length of 3’-sticky overhang	9 bases (CGCTTGTCA)	10 bases (CGGACGGCGC)	N/A	10 bases (CGGACGGCGC)	10 bases (CGGTCGGTTA)	11 bases (CTCGTAGGCAT)
